# Resource Review: ActivePresenter v9

**DOI:** 10.5195/jmla.2023.1731

**Published:** 2023-10-02

**Authors:** Stephanie M. Swanberg

**Affiliations:** 1 sswanberg@msp.edu, User Services Librarian, Moustakas Johnson Library, Michigan School of Psychology, Farmington Hills, MI.

## Abstract

**ActivePresenter v9**. Atomi Systems, Inc, Headquarters: No 281, Group 1, Phu Dien ward, North Tu Liem, Ha-noi, Vietnam; support@atomisystems.com; https://atomisystems.com/download/; $0 - $399 depending on license selected. Technical requirements: Operating Systems: Windows 8.1, Windows 10, Windows 11 (only 64-bit is supported), macOS 10.15, macOS 11, macOS 12; Hardware: 2 GHz or faster processor with SSE2; greater than 4 GB of RAM; 4 GB of available hard-disk space for installation; microphone.

While there are a variety of free applications and software available for video recording and/or editing, ActivePresenter provides a single authoring tool for developing online learning objects presented in a familiar interface. For the purposes of this review, online learning objects are broadly defined as: “digital materials…with defined instructional value that can be used in a variety of ways to improve teaching and learning” [1]. This is inclusive of videos, tutorials, modules, webpages, and more that are developed with specific learning objectives in mind. ActivePresenter is a desktop-based application available for both macOS and Windows. Of interest, administrative access is needed to initially install ActivePresenter and deploy any future updates.

ActivePresenter has three versions: Free ($0), Standard ($199), and Pro ($399). The free version can be downloaded to multiple devices and is intended for non-commercial use only. The Standard and Pro version licenses cover a single download for one device and includes perpetual access to the purchased version as well as any future software updates. Additionally, there is an educational discount program offering reduced individual Pro licenses ($199) and discounts for Pro educational licenses purchased in bulk.

The free version of ActivePresenter is surprisingly robust for screen capture/video recording, editing, and video publishing options. The following sections will detail each of these three areas.

## SCREEN CAPTURE/VIDEO RECORDING

Like other screen recording tools, ActivePresenter allows users to record the full screen or adjust the recording area to the desired size. The microphone can also be turned on or off providing the flexibility to either record audio live or add narrations later during the editing process. If a camera is available, it can be turned on/off to include a talking head with the screen capture or just to record the screen only. In addition, ActivePresenter imposes no limits on the number of videos that can be recorded. There are also no time limits on the length of the recording, compared to other free video recording tools, such as Loom or ScreenPal (formerly Screen-cast-O-Matic), that do impose time limits.

**Figure 1:**
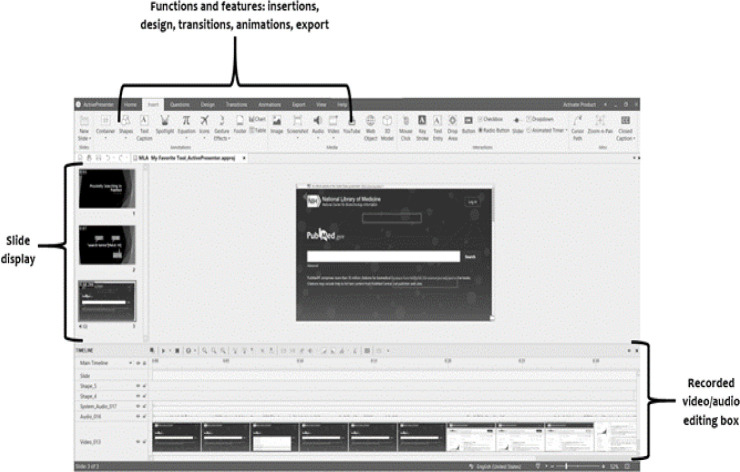
ActivePresenter interface

## VIDEO & AUDIO EDITING AND LEARNING OBJECT CREATION

The major advantage to using ActivePresenter is that it has a simple learning curve. ActivePresenter mimics the Microsoft Office layout so no matter the level of experience with video editing, the user can easily recognize the layout and find their way around the product. Once a video is recorded, it is opened in the ActivePresenter interface for editing. The figure below highlights the major areas of the ActivePresenter interface.

The video and audio editing features included in the free version are: cutting/copying/deleting sections of recorded video/audio, splitting or combining video/audio files, importing other video/audio files, and inserting closed captioning. Closed captions have to be manually entered in the free version, so it is recommended that videos be uploaded to YouTube or other sharing platforms to utilize their automatic speech-to-text closed captioning.

ActivePresenter can also be used to create professional online learning objects by combining screencast/video recordings and PowerPoint slides. The recording is treated as a single “slide” in ActivePresenter as if in the PowerPoint environment. Existing PowerPoint files can be imported into ActivePresenter or new slides can simply be added to accompany the video, such as an introduction and ending slide. In addition, other familiar PowerPoint features are included such as adding shapes (e.g., call-out boxes, arrows), inserting animations and transitions between slides (e.g., zoom-ins/outs, etc.), changing slide layout and colors, and more (example video [2]). Just note that all of the features from the Standard and Pro versions are visible, but not enabled in the free version.

## VIDEO PUBLISHING

For video publishing, the free version of ActivePresenter has two options: 1) exporting to an image file, or 2) exporting to a video file (MP4, WEBM, MKV, AVI, WMV). These two options do not include a watermark, however those may appear when attempting to export file types from the free version that are only available in the Standard or Pro versions (e.g. HTML 5 preview). The exported video file can then be uploaded to YouTube or another platform for publishing. For librarians that casually create video tutorials, the free version of ActivePresenter is likely sufficient for recording and editing needs. However, the Pro version should be considered for librarians who frequently develop learning objects and online tutorials, or record lectures. In particular, if the intent is to integrate content into learning management systems, the Pro version includes the ability to export learning objects to HTML 5 and to Sharable Content Object Reference Model (SCORM) 1.2, SCORM 2004, and xAPI. For a comparison of the features between the three versions, visit their website [3].

Two comparable products to ActivePresenter that offer screen recording, video editing, and publishing in a single authoring tool regardless of operating system are ScreenPal (previously Screencast-O-Matic) and Camtasia [4, 5]. As iMovie is only available to use on Apple devices, it is not included in this review [6]. Overall, these three tools offer a similar product with comparable features, so choosing which one to use will depend on individual preferences, device compatibility, and cost. Table 1 provides a comparison of the features of the three tools as of Spring 2023. Of the two products with free versions, ActivePresenter and ScreenPal both offer robust recording, editing, and publishing capabilities that can easily support librarians who occasionally create online learning objects. Again, the most distinguishing characteristic of ActivePresenter is the familiar Microsoft Office-like interface and compatibility with Microsoft PowerPoint. This gives it wide appeal as it can be adopted quickly and at no cost.

**Table 1:** Comparison of ActivePresenter, ScreenPal, and Camtasia

Feature		ActivePresenter *(free version)*	ScreenPal *(free version)*	Camtasia
	Web- or desktop-based application?	Desktop-based	Web-based	Desktop-based
	Operating system compatibility	Windows, macOS	Windows, macOS, Chromebook	Windows, macOS
**Pricing**				
	Price Range	$0-$399 (single user license)	$0-$120 annually (single user)	$299.99 (single user license)
	Educational discount available?	✓	✓	✓
	Multi-user/bulk purchase discount available?	✓	✓	✓
**Screen Capture/Video Recording Features**				
	Recording time limits	—	15 minutes	—
	Unlimited video recordings	✓	✓	✓
	Adjustable screen recording area	✓	✓	✓
	Webcam video capture available	✓	✓	✓
**Video & Audio Editing Features**				
	Trim recordings (cut, copy, delete)	✓	✓	✓
	Add shapes, animations, transitions	✓	✓	✓
	Add tables, charts, figures	✓	—	—
	Import music and other audio files	✓	✓	✓
	Import Microsoft PowerPoint files	✓	—	✓
	Add quizzes or other testing features	Available in paid versions only	Available in paid versions only	✓
	Add closed captioning	Manually add *(Automated speech-to-text captioning available in paid versions)*	Manually add *(Automated speech-to-text captioning available in paid versions)*	Manually add *(Speech-to-text captioning available in Windows version only)*
**Publishing and Storage**				
	Watermark on learning objects	Depends	✓	—
	Local or cloud storage?	Local only	Both available depending on plan	Local only
	Storage limits	Storage only limited by the local memory available on individual device	Cloud storage limits depend on paid plan	Storage only limited by the local memory available on individual device
	Export to video file	✓	✓	✓
	Export to HTML5	Pro only	—	✓
	Export to SCORM for LMS integration	Pro only	✓	✓
	Share via social media	—	✓	—
	Upload directly to YouTube	Manual upload only	✓	✓

ActivePresenter is a recommended tool for librarians and information professionals who occasionally or regularly develop online learning objects including instructional tutorials, modules, and lectures, as well as embed learning objects into web pages or learning management systems. Depending on the extent of work in creating these materials, a paid version to ActivePresenter might be more appropriate to consider. Beyond libraries, it can also be recommended to faculty for use in online teaching, or students for class projects.
